# Microbial community profiling and culturing reveal functional groups of bacteria associated with Thai commercial stingless worker bees *(Tetragonula pagdeni*)

**DOI:** 10.1371/journal.pone.0280075

**Published:** 2023-03-01

**Authors:** Chainarong Sinpoo, Ammarin In-on, Nuttapol Noirungsee, Korrawat Attasopa, Panuwan Chantawannakul, Veeranan Chaimanee, Patcharin Phokasem, Tial Cung Ling, Witoon Purahong, Terd Disayathanoowat

**Affiliations:** 1 Bee Protection Laboratory, Department of Biology, Faculty of Science, Chiang Mai University, Chiang Mai, Thailand; 2 Bioinformatics & Systems Biology Program, King Mongkut’s University of Technology Thonburi (Bang Khun Thian Campus), Bang Khun Thian, Bangkok, Thailand; 3 Department of Entomology and Plant Pathology, Faculty of Agriculture, Chiang Mai University, Chiang Mai, Thailand; 4 Department of Agro-Industrial Biotechnology, Maejo University Phrae Campus, Rong Kwang, Phrae, Thailand; 5 Department of Soil Ecology, UFZ-Helmholtz Centre for Environmental Research, Halle (Saale), Germany; City University of New York, The City College of New York & CUNY Graduate Center, UNITED STATES

## Abstract

Stingless bees play a crucial role in the environment and agriculture as they are effective pollinators. Furthermore, they can produce various products that can be exploited economically, such as propolis and honey. Despite their economic value, the knowledge of microbial community of stingless bees, and their roles on the bees’ health, especially in Thailand, are in its infancy. This study aimed to investigate the composition and the functions of bacterial community associated with *Tetragonula pagdeni* stingless bees using culture-independent and culture-dependent approaches with emphasis on lactic acid bacteria. The culture-independent results showed that the dominant bacterial phyla were Firmicutes, Proteobacteria and Actinobacteria. The most abundant families were Lactobacillaceae and Halomonadaceae. Functional prediction indicated that the prevalent functions of bacterial communities were chemoheterotrophy and fermentation. In addition, the bacterial community might be able to biosynthesize amino acid and antimicrobial compounds. Further isolation and characterization resulted in isolates that belonged to the dominant taxa of the community and possessed potentially beneficial metabolic activity. This suggested that they are parts of the nutrient acquisition and host defense bacterial functional groups in Thai commercial stingless bees.

## Introduction

Stingless bees (Apidae: Meliponini) are a group of eusocial insects that play a pivotal role in pollination [1], particularly wildflowers in most tropical and subtropical areas [2]. Their social systems and division of labor are similar to honeybee colonies [3]. They also have the ability to produce bee products, such as honey, beebread, and propolis [1, 4].

The study of bee gut microbiota has received increasingly much attention, since gut microbiota play essential parts in improving the host’s quality of life. Two core functional groups include nutrient metabolism and defense functional groups. In particular, the lactic acid bacteria found in the gut of honeybees can protect the host from infections by colonizing and secreting antagonistic substances [5–7]. The bacterial gut communities of corbiculate bees are made up of highly conserved core bacteria, including *Snodgrassela alvi*, *Gilliamella apicola*, *Bifidobacterium asteroides*, *Lactobacillus* Firm-4, and *Lactobacillus* Firm-5 [6, 8–11]. However, there are only a limited number of studies on gut microbiota of stingless bees. Suphaphimol et al. (2020) found that Proteobacteria and Firmicutes were predominantly abundant among *Lepidotrigona terminata* [12]. Recently, Tang et al. (2021) analyzed the bacterial communities from stingless bees in China found that the dominant bacteria genera were Acetobacter-like bacteria, *Snodgrassella*, *Lactobacillus*, *Psychrobacter*, *Pseudomonas*, and *Bifidobacterium* [13]. Most culturable genera of bacteria associated with stingless bees are from *Bacillus*, *Streptomyces*, and *Lactobacillus* [14–16].

Lactic acid bacteria (LAB) such as *Lactobacilli* and *Bifidobacterium* are the most common genera of LAB that are the most abundant genera in the gut of honeybees [7, 17–19]. *Lactobacillus* was also commonly found in the gut of adult honeybees (*Apis mellifera*) [18, 20, 21]. *Lactobacillus* and *Bifidobacterium* genera were abundant in stingless bees from China [13]. These bacterial group can enhance stingless bees digestion and nutrient acquisition from honey, beebread, and propolis [22]. Other than that, *Lactobacillus* has a beneficial influence on immunity [23, 24], as it can secrete antimicrobial compounds, such as bacteriocins and lactic acid, to protect their hosts [25, 26]. Forsgren et al. (2009) revealed that LAB isolated from honey bees showed an antagonistic effect in the in vitro growth of *Paenibacillus larvae*, which causes American foulbrood (AFB) in honey bees and their infection under in vivo conditions [13]. Suphaphimonl et al. (2020) reported that *F*. *fructosus* suppressed pathogen growth [12]. *Bifidobacterium* has an essential role as the degrader of hemicellulose and pectin in honeybees’ guts [11].

Studies of the bacterial community of Thai stingless worker bees, especially *T*. *pagdeni*, are limited. Previous studies reported only on microbial identification but not their functions [13, 27–29]. This study complements the previous report by determining both the diversity and the function of the bacterial communities associated with *T*. *pagdeni*, using culture-independent methods, as well as, culture-dependent methods in order to identify and characterize bacterial microbiota associated with Thai commercial stingless worker bees.

## Materials and methods

### Specimen collection and species identification

The specimens of stingless bees were collected from a total of six colonies in Chiang Mai Province, Thailand. They were transferred directly into absolute ethanol and preserved at -20°C until the DNA extraction step. The coordinates and altitudes of the discovered nests were recorded according to GPS data (Garmin). The hives’ locations are listed in S1 Table. The ethical consent was waived from Chiang Mai University for this study because the specimens were invertebrates.

The specimens were pinned and preserved as dry specimens to examine their morphological features, and taxonomically identify the specimens. The identification was performed according to the original descriptions and identification keys from Schwarz (1939) and Sakagami (1978) [30, 31]. The worker morphology was examined under a Nikon SMZ1500 stereomicroscope (from PCYU, Canada). The morphological diagnostic characteristics were described. The morphological terminology and abbreviations follow Michener (2007) [32]. Some of the important diagnostic characteristics were magnified and photographed under a microscope.

To confirm the taxonomic identification, molecular identification was performed. One of the typical standard methods for species identification of bees is using mitochondrial cytochrome oxidase I (COI) gene. Stingless bee samples were sterilized following the method of Pakwan et al. (2018) [33]. The genomic DNA of one individual per nest was extracted using the DNAeasy Blood & Tissue Kit (Qiagen, USA) following the manufacturer’s instructions. The DNA concentration of each sample was measured using a Nanodrop spectrophotometer (Thermo Scientific, USA). The genomic DNA samples were stored at -20°C until they were needed as a DNA template for the PCR (polymerase chain reaction). The PCR amplification of approximately 685 base pairs of COI genes was performed by using the forward primer LCO1490 (5’-GGTCAACAAATCATAAAGATATTGG-3’) and the reverse primer HC02198 (5’-TAAACTTCAGGGTGACCAAAAAATCA-3’) [34]. PCR components were 25 μl containing 2 μL of DNA extract, 12.5 pM of each primer, 0.2 mM of each dNTP, 0.2 mM MgCl2, 1X reaction buffer and 2.5 units of Taq DNA polymerase (Invitrogen). The PCR conditions were 94°C for 1 min (initial denaturation step), five cycles of 94°C for 1 min, 50°C for 1.5 min, 72°C for 1 min, 35 cycles of 94°C for 1 min (denaturation step), 50°C for 1.5 min (annealing step), 72°C for 1 min (extension step), and a final extension of 72°C for 5 min. 1 μL of product was mixed with 6X loading dye (New England BioLabs, USA) then loaded onto 1% agarose gel. The size of the fragments of DNA was compared with a 100 bp DNA ladder (New England Biolabs, USA) by agarose gel electrophoresis. The electrophoresis ran for 40 min at 100 V with TAE buffer. The loaded gel was stained by ethidium bromide and visualized by UV light. PCR products were purified using a PureLink Quick PCR Purification Kit (Invitrogen, Lithuania, USA) following the manufacturer’s protocol. DNA sequencing was performed and automatically determined in a genetic analyzer (Macrogen Inc., South Korea).

### Bacteria associated with stingless bees

#### Culture-independent approach: Illumina MiSeq and bioinformatic analysis

Surface sterilization of the stingless bees was conducted prior to genomic DNA extraction as described above. The total genomic DNA of the microbiota were extracted and a total weight of 0.1 grams of the stingless bees from six colonies were sampled using the ZymoBIOMICS DNA Miniprep Kit (ZYMO Research, Germany and EU) and eluted by Tris–EDTA buffer. Then the concentration and purity of the genomic DNA were determined with a Nanodrop spectrophotometer (Thermo Scientific, USA) using 1 μl at 260 nm. The genomic DNA samples were stored at -20°C until use. The bacterial 16S rRNA hypervariable V3−V4 regions were used to identify bacteria with the primers 341F (341F: 5’-CCTAYGGGRBGCASCAG-3’) and 806R (5’-GGACTACNNGGGTATCTAAT-3’) containing the specific barcode sequence that was used for the amplification of the V4 regions. The amplicon DNA was sequenced with an Illumina MiSeq sequencer (Seoul, South Korea). Raw sequences were preprocessed by using fastp [35], and the adaptor sequences and low-quality reads (phred score > 30) were removed in this step. QIIME2 version 2020.8 [36] was then used for further data preprocessing and processing. Then, the data were denoised to ASVs (amplicon sequence variants) using DADA2 [37], and chimeric sequences were excluded in this step. The ASV table was rarefied before diversity analysis and taxonomic identification, and was classified against the Greengenes database (available on http:/greengenes.lbl.gov) [38]. Then, the taxonomic identification was refined by using the HoloBee database [39], and the bi-directional BLAST was performed with cut-off at 99% identity. For the downstream analysis, bacterial community functions were predicted, analyzed, and visualized by FAPROTAX (Version 1.2.4) [40], PICRUSt 2.3.0b [41], and Metacoder [42]. Multiple sequence alignments and phylogenetic tree constructions were carried out in webPRANK [36]. The tree was exported as a Newick file. Then, ele3, which is based on Python 3.8, was used for visualizing the tree from the Newick file. The top ten most abundant taxa from MiSeq and full length 16S sequences of isolates were refined according to their taxonomic identification in HoloBee database [39] using bidirectional BLAST with the percent identity cut-off at 99% in both directions of BLAST.

#### Culture-dependent approach: Isolation and characterization of functional groups

Surface sterilization was carried out as described previously [33]. The abdomens of 30 individual samples (total weight of 0.1 grams) were removed with scissors and placed in a 1.5 ml microcentrifuge tube. Then, these were homogenized with 900 μl of 0.85% (v/v) NaCl solution. The suspensions were serially diluted with a tenfold dilution using 0.85% (v/v) NaCl solution from the concentration of 10^−1^ to 10^−5^, as well as 100 μl solutions spreading to petri dishes containing 25 mL de Man–Rogosa–Sharpe agar (MRS) to select for lactic acid bacteria. The plates were incubated for 48 hours at 30°C. Subsequently, LAB colonies were counted in a colony forming unit (CFU g^-1^ sample). All assays were performed in triplicates to allow the calculation of the mean and standard deviation. Molecular methods were used to confirm the LAB isolates. The genomic DNA was extracted from LAB isolates using the ZymoBIOMICS DNA Miniprep Kit (ZYMO Research, Germany and EU). DNA extraction was performed following the manufacturer’s protocols. In order to identify the bacterial isolates, the 16S rRNA gene was amplified using two universal primers (27F, 5’-AGAGTTTGATCMTGGCTCAG-3’ and 1492R, 5’-GGYTACCTTGTTACGACTT-3’) [43]. PCR amplification was performed in a total volume of 25 μl containing 2 μL of DNA extract, 12.5 pM of each primer, 0.2 mM of each dNTP, 0.2 mM MgCl2, 1X reaction buffer and 2.5 units of Taq DNA polymerase (Invitrogen) under the following conditions: 95°C for 5 min (initial denaturation step), 30 cycles of 95°C for 30 sec (denaturation step), 53°C for 2 min (annealing step), 72°C for 2 min (extension step), and the final extension of 72°C for 10 min. All bacterial sequences were trimmed using Mega 7 (7.0.14) and aligned in BioEdit 7 (7.1.3.0). These sequences were identified using BLASTN in GenBank. Similar bacterial sequences were collected and used to build a phylogenetic tree with FastTree using a GTR model [44]. To determine the presence of enzymes and the functional roles of LAB isolates in promoting *T*. *pagdeni* health, the lipases and amylases activity assays were performed as previously described [20].

## Results

The bacterial communities associated with *T*. *pagdeni* from Northern Thailand were investigated, using culture-dependent and culture-independent approaches. For the culture-dependent study, ten individuals (with a total weight of 0.1 grams) from each colony of *T*. *pagdeni* (n = six nests) were collected as living specimens in Chiang Mai University, Chiang Mai, Thailand.

### Taxonomy of *T*. *pagdeni*

**Fig 1:**
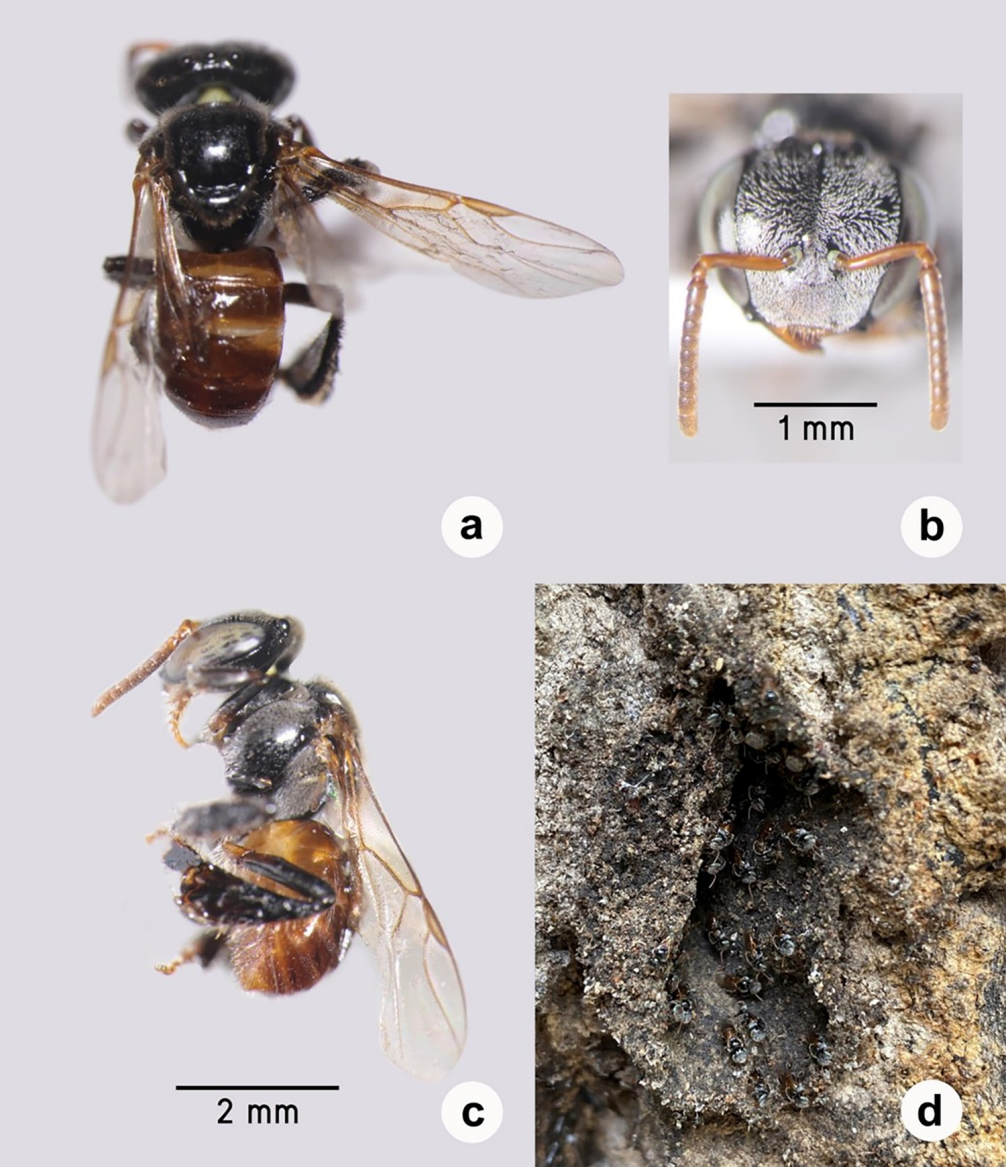
*T*. *pagdeni* worker bee: dorsal habitus (A), frontal view (B), lateral habitus (C) and their nest entrance (D).

The stingless bee specimens were identified as *T*. *pagdeni* using the identification key of Schwarz (1939) and Sakagami (1978) [30, 31]. Their structures were measured according to a range (Average±SD, n = 12) as follows: a body length of 4.33–4.85 (4.576±0.155) mm, a head width of 1.72–1.75 (1.737±0.015) mm, and wing length of 1.05–1.14 (1.095±0.03) (Fig 1).

DNA was extracted and the COI gene sequence was amplified successfully from individual stingless bee specimens from six localities. All the sequences were 658 base pairs long after removing the primer from both ends. We found a strong A+T bias in the COI gene barcoding from the mtDNA (S1 Fig). All new sequences were deposited in GenBank

#### Analysis of the Illumina NGS data

The total number of bases, reads, GC (%), Q20 (%), and Q30 (%) were calculated, and quality-filter raw fastq files from the six samples, with an average sequence length of 250 bases. The quality scores of the reads were averagely good (Q30). A total of 400,047 high-quality bacterial sequences were obtained from the Illumina sequencing of six colonies of *T*. *pagdeni*, with 2427 bacterial amplicon sequence variants (ASV) identified at the 97% cut-off level.

#### Bacterial communities in the gut of *T*. *pagdeni*

**Fig 2:**
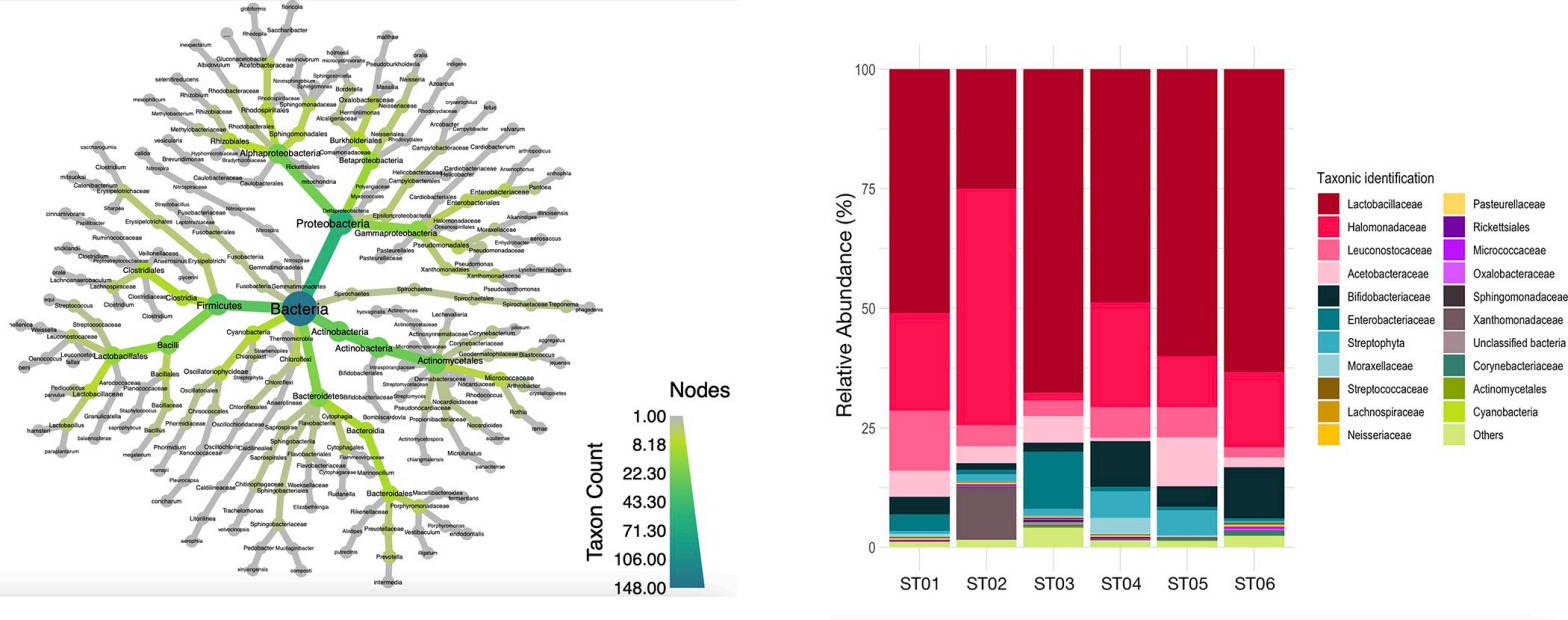
The taxonomic composition at phylum and genus levels. (a) Heat tree of the abundance of bacterial taxa at different ranks present in the *T*. *pagdeni* stingless bees that were determined using culture-independent approach. The size and color of the nodes and edges are correlated with the abundance of taxa. The central nodes are the total of all the other nodes in the tree for each phylum [42]. (b) The gut bacterial composition of *T*. *pagdeni* stingless bees from each colony. The columns represent a sample, the colors show different families, and the length of the column represents the relative proportion.

The taxonomic classification of 18 bacterial phyla was detected in total. We found four phyla that had an abundance higher than 1%. Approximately 60% of the sequences were classified as Firmicutes. While Proteobacteria accounted for ~30% followed by Actinobacteria at 6%. (Fig 2A, S3 Fig, S3 Table). The redundant taxonomies of three replicated data of three samples were collapsed to their parents at the lowest genus level. The top 12 abundant bacteria were collapsed taxa of each sample. The amounts of all samples were normalized before bar plotting. The figure was ranked according to the sum number of taxa from all samples. Bacterial communities in the gut of *T*. *pagdeni* comprised 12 major taxa (Fig 2, S4 Table): genera of Lactobacillaceae excluding *Lactobacillus* (30.59%± 11.02%), *Lactobacillus* (20.81%± 15.52%), and Halomonadaceae *(*20.09%± 16.29%), *Leuconostoc* (5.65%± 3.96%), *Bombiscardovia* (5.28%± 3.96%), *Saccharibacter* (4.5%± 3.37%), *Pantoea* (2.53%± 4.69%), and Xanthomonadaceae (1.84%± 4.49%). *Alkanindiges*, *Pediococcus* and Enterobacteriaceae accounted for less than 1% of the bacterial communities.

#### Functional prediction among bacterial communities

**Fig 3:**
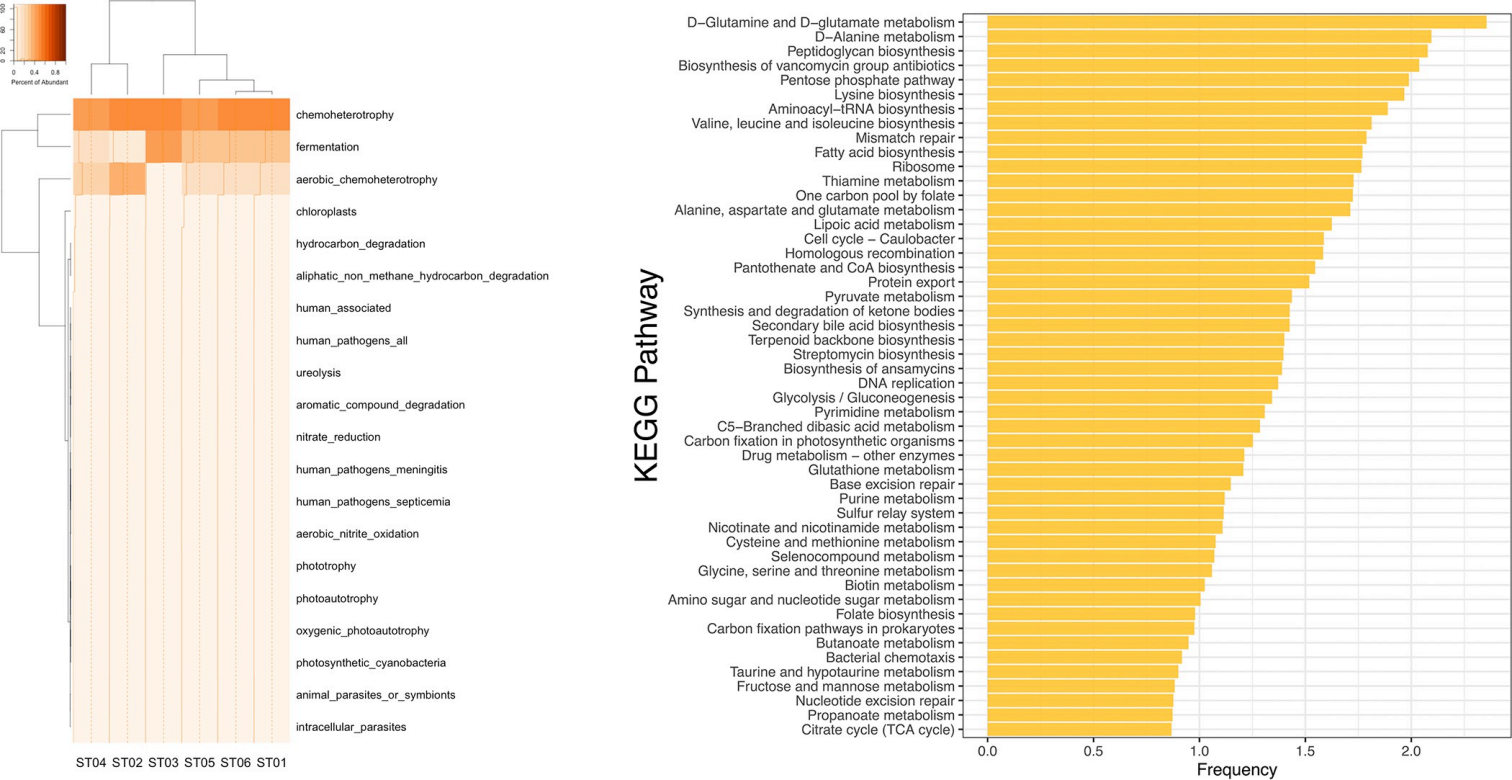
Bacterial functional gene prediction analysis from the gut of *T*. *pagdeni*: (a) FAPROTAX based on the relative abundance of bacterial taxa. (b) KEGG pathway entries with the top 50 ranking from the most frequent to the least.

Bacterial functions were predicted using FAPROTAX based on the relative abundance of bacteria. Overall, 38 functional groups were identified in the bacterial communities (Fig 3A). The dominant functional genes of *T*. *pagdeni* are those categorized in chemoheterotrophy, fermentation, aerobic chemoheterotrophy, human pathogens, animal parasites and symbionts. The functional prediction was performed using PICRUSt 2.3.0b, then the top 50 most frequent were visualized as shown in Fig 3B. The bacterial genes that increased in functional activity were genes involved in D-glutamine and D-glutamate metabolism, D-alanine metabolism, peptidoglycan biosynthesis, the biosynthesis of vancomycin group antibiotics, the pentose phosphate pathway, and lysine biosynthesis.

#### Lactic acid bacteria isolation, identification, and characterization

The highest numbers in the bacterial population were at most 1.66±0.03× 10^4^ CFU g^-1^ and at least 1.46±0.04× 10^4^ CFU g^-1^. The 12 LAB isolates were categorized into four genera of the phyla Firmicutes including *Enterococcus*, *Bacillus*, *Leuconostoc*, and *Weissella*. The isolates had the 16S rRNA sequence with more than 98% identity to *Weissella hellenica* (six isolates), *Enterococcus faecalis* (two isolates), *W*. *bombi*, *Bacillus cereus*, *Leuconostoc citreum*, and *L*. *mesenteroides*. The estimation of the phylogenetic relationship of LAB isolation was calculated using neighbor-joining, and *Clostridium botulinum* (accession no.: JFGN01000349) were used as an out group (S2 Fig). Among the LAB isolates from the six hives, only *Bacillus* sp. (CMU-LAB06) was found to produce lipases and amylase under the conditions used in this investigation.

**Fig 4:**
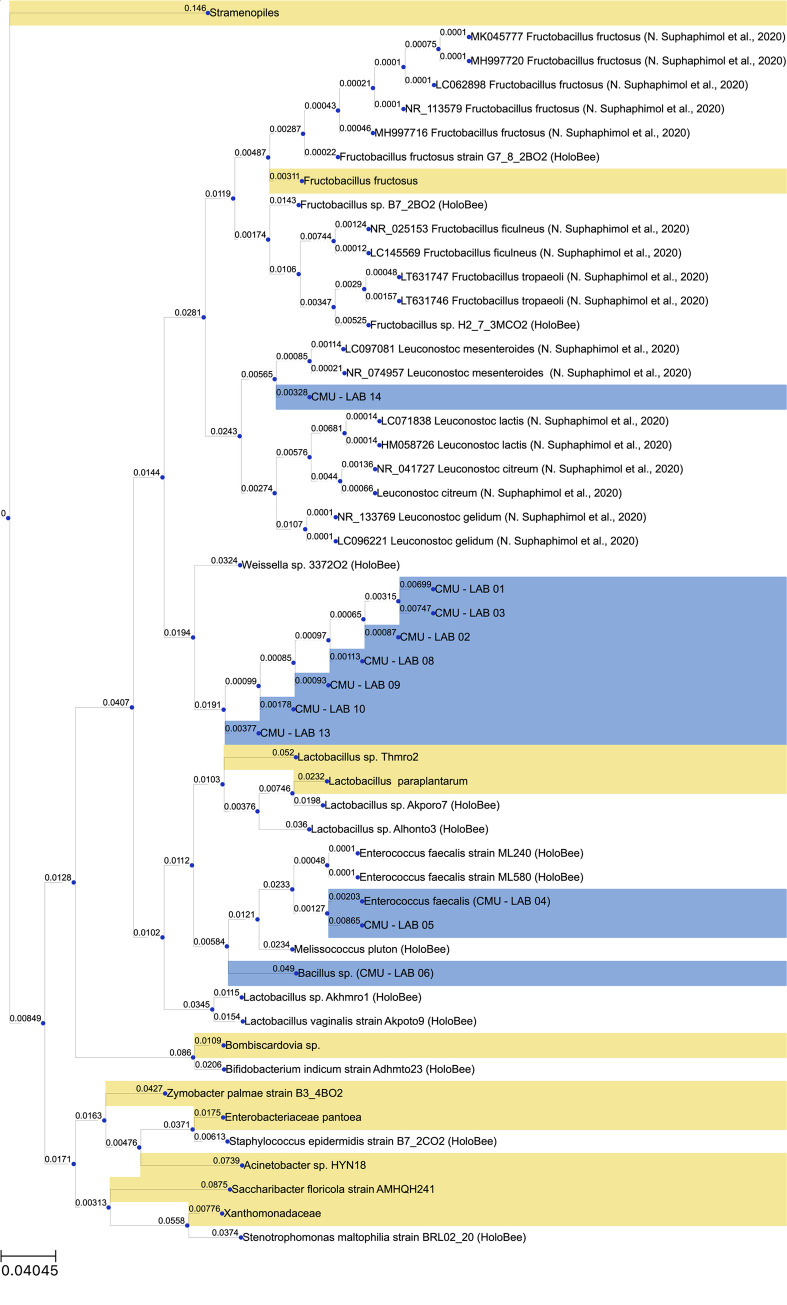
The phylogenetic tree showing the dominant bacterial phyla and isolates. The sequences labeled in yellow are the top ten most abundant bacteria detected by amplicon sequencing in this study. The sequences labeled in blue are the sequences of bacterial isolates from this study.

To determine whether the isolates belong to the functional groups as those found in community profiling, the phylogenetic tree containing the 16S sequences from Illumina MiSeq and isolates were constructed. The phylogenetic tree is composed of the top ten most abundant taxa obtained from Illumina MiSeq sequencing (yellow) and the culture-dependent approach (blue), as well as 16S rRNA gene from the literatures, and the HoloBee database, which is an archive of microorganisms associated with honeybees (*Apis* sp.) [45]. *Weisella* (CMU-LAB 01–03 and 08–13), *Enterococcus* (CMU-LAB 05 and 06), and *Bacillus* (CMU-LAB 04) isolates were grouped with sequences obtained from the HoloBee database (Fig 4). While, *Leuconostoc* isolates (CMU-LAB 14) were grouped among *Leuconostoc* previously isolated from another species of stingless bee (*L*. *terminata*) [12].

## Discussion

We used culture-independent sequencing (Illumina MiSeq) to describe the bacterial communities associated with *T*. *pagdeni* from Northern Thailand. The data from high-throughput sequencing (Illumina MiSeq) were analyzed to determine the composition of gut-dominant bacteria. Our findings showed that the dominant bacterial phyla were Firmicutes, Proteobacteria, and Actinobacteria. The dominant bacterial phyla were similar to those of *T*. *pagdeni* from China. However, the relative abundance of Proteobacteria in our study constitute to the lower extent compared to the previous study (82.46%) [13]. Interestingly, the proportions of Proteobacteria were more similar to those of *A*. *melliflora* (42.16%) [46]. Actinobacteria contributed higher relative abundance than previously reported in *T*. *pagdeni*, and *A*. *melliflora* [13, 46]. The divergence of bacterial compositions associated with bees could be due to various factors including season, food sources, and health status of bee [47, 48]. Therefore, the bacterial composition reported in our study could be unique to *T*. *pagdeni* from Northern Thailand.

The most dominant class in Firmicutes was Bacilli. The members of this class found in this study included *Lactobacillus*, *Leuconostoc*, and *Pediococcus*. The most dominant family of Actinobacteria was Bifidobacteriaceae. The genera found in this study were *Bombiscardovia* and *Bifidobacterium*. *Lactobacilli* and *Bifidobacterium* are the most abundant genera in the gut of honeybees [7, 17–19, 21], and stingless bee [13]. These groups of bacteria has been reported to enhance stingless bees digestion, nutrient acquisition, and immunity [22–26].

The major gamma-proteobacteria belonged to Halomonadaceae. which has been reported to show elevated abundance in bees treated with antibiotic [49]. The exposure of stingless bees in our study to antibiotic was unlikely, since they were collected from natural habitat. Alternatively, Halomonadaceae are often present in low-water activity environments, it might thrive in sugar-rich environments such as those found in beehives. One of the major genera of Halomonadaceae identified in our study was *Zymobacter* (S4 Table). A member of this genus has been isolated from palm sap [50]. Food sources of stingless bee in Thailand also include pollens of plants in Palmae family such as bitter nut and coconut [1]. Therefore, these microbes might become a part of *T*. *pagdeni* microbial community through ingestion of Palmae plant pollens. Whether the members of this family are unique in Thai stingless bee or contribute to their health status needs further investigation.

Results from functional assignments elucidated chemoheterotrophy and fermentation as the essential functions of bacteria associated with *T*. *pagdeni* (Fig 3A). Intuitively, the bacteria residing in the gut of insects mostly receive carbon and energy through the oxidation of monosaccharides (glucose, galactose, fructose) and disaccharides (lactose, sucrose), which are derived from bees diets [51]. Firmicutes (Bacilli), Proteobacteria (beta-proteobacteria, alpha-proteobacteria, gamma-proteobacteria), Actinobacteria, Cyanobacteria, and Bacteroidetes are the major executors of chemoheterotrophy and aerobic chemoheterotrophy. The bacteria, in turn, provide nutrients and possibly amino acids to hosts via the fermentation of indigestible food. Undigested carbohydrates are mostly fermented to SCFAs (such as butyrate and acetate) and gases (hydrogen, carbon dioxide, methane, and hydrogen sulfide) in the anaerobic environment inside the gut [52].

The functional roles of bacteria were mostly involved in amino acid metabolisms and secondary metabolites biosynthesis (Fig 3B). We found abundant genes related to the D-glutamine and D-glutamate biochemical metabolic pathway. The glutamate metabolism is closely related to the process of ammonia assimilation [53], and plays an essential role in response to acid stress and other stresses that were found in Proteobacteria [54]. Glutamine is the an amino acid that participates with key enzymes for recycling nitrogenous waste products, glutamine–glutamate synthase, and glutamate dehydrogenase which were found in alpha-proteobacteria, namely *Bartonella* [55], as well as [56], *Lactobacillus reuteri* [54].

The genes related to biosynthesis of vancomycin were predicted. This antibiotic group can protect the insect hosts from pathogenic infections [23, 57–60], especially from Gram-positive bacteria such as *P*. *larvae* and *Melisococcus plutonius*, the causal agents of American foulbrood (AFB) and European foulbrood, respectively [58, 61]. Beneficial microbiota such as *Lactobacillus*, *Leuconostoc*, *Pediococcus*, and *Weisella*, are, on the other hand, intrinsically resistant to vancomycin [62, 63]. This is congruent with results from both culture-independent and culture-dependent approaches in our study, as *Lactobacillus*, *Leuconostoc*, *Pediococcus* and *Weissella* were found to be associated with *T*. *pagdeni*. Moreover, Halomonadaceae has been reported to resist vancomycin under specific condition [64]. The presence of vancomycin could be the cause of high relative abundance of Halomonadaceae in *T*. *pagdani*. The distinctively high abundance of Actinobacteria found in our study could result in elevated production of vancomycin, which prevent colonization of pathogens, while preserve growth of functional groups beneficial to stingless bees. The role of autochthonous production of vancomycin in regulating gut microbiota of *T*. *pagdani* worker bees warrant further investigation.

The pentose sugar D-xylose, which are found in the nectar sugar of some plants, is a structural component of plant cell walls. The metabolism of gut bacteria is related to breaking down the hexoses (C6) and pentoses (C5) of dietary fibers and inducing xylose-metabolizing bacteria to produce short-chain fatty acids (SCFAs) [65]. This was previously reported as the main pathway for lactic acid production from xylose associated with the pentose phosphate (PP)/glycolytic pathway in the strain *Enterococcus mundtii* QU 25 [66]. Moreover, *Lactobacillus* spp. has numerous phosphotransferase systems linked to the uptake of sugars, while *Bifidobacterium* has genes for carbohydrate utilization [67, 68]. These bacteria can utilize glucose and fructose, the most abundant source of sugars in honeybee food [69].

The conventional culture in this study showed that a common group of LAB found in *T*. *pagdeni* was Firmicutes. Most of the isolates were *Weissella* and *Leuconostoc*. *Weissella* isolates were grouped among the sequences recovered from *Apis* sp. This suggested that *Apis* and *Tetragonula* might share this microbial functional group. *Leuconostoc* sp. isolated from *T*. *pagdeni* were clustered with *Leuconostoc* sp. isolated from another stingless bees *L*. *terminata* in Thailand [12]. Therefore, this bacterial functional group might be unique to stingless bee in Thailand. The roles of these bacterial functional groups and the extent to which they contribute to stingless bee health, are yet to be determined. We also isolated and identified *B*. *cereus* in this study. Consistent with previous studies, bacterial species with *B*. *cereus* strains were isolated from honeybees [14, 70], solitary bees [22, 71], and stingless bees [15, 27]. *B*. *cereus* CMU-LAB04 can produce lipases and amylase, consistent with most previous studies on stingless bees (*Heterostrigona itama*), *Bacillus* species have been shown to exhibit proteolytic, lipolytic, and cellulolytic activities [27]. The enzymatic reactions by these bacterial isolates might play an essential role in the digestion of carbohydrates from nectar and proteins presented in pollen and nest product [27, 72].

We have described the bacterial communities associated with *T*. *pagdeni* from Northern Thailand using a combination of culture-independent approach and culture-dependent approach. The taxonomic profiling showed that the major groups of bacteria belonged to the family Lactobacillaceae, Halomonadaceae and Bifidobacteriaceae. The main metabolic pathways were amino acid metabolisms, antimicrobial productions, and fermentation probably required for colonizing and preventing ingrowth of stingless bee pathogens. We were able to isolate *Weissella*, *Leuconostoc*, and *Bacillus*, which were the core functional groups of Thai stingless bee and might play vital role in maintaining stingless bee health. Our work provided the basis for the studies of insect-microbes interactions in Thai commercial stingless bees, and paved the way to application and manipulation of microbes in pollinator conservation, apiculture, and ultimately, food security.

## Supporting information

S1 Fig A phylogenetic tree showing the relationship of cytochrome oxidase subunit I (COI), partial cds; mitochondrial from native stingless bees (*Tetragonula pagdeni* Smith) collected in Northern Thailand. The tree was built using the Maximum Likelihood method. The sequences of *B*. *terrestris*–KT074036 was used as an outgroup to the tree. Numbers at each node represent bootstrap values as percentages of 1000 and only bootstrap greater than 70% are shown.
(TIF)

S2 Fig Estimation of phylogenetic relationship of lactic acid bacteria isolation using neighbor-joining. The sequences of *Clostridium botulinum*–JFGN01000349 was used as an outgroup. Numbers at each node represent bootstrap values as percentages and only bootstrap values greater than 70%.
(TIF)

S3 Fig The most abundant phyla with bacterial communities associated with *Tetragonula pagdeni*. (TIF)

S1 Table Material used in the phylogenetic analysis with the sample localities, and name of the collector. (PDF)

S2 Table Isolation and characterization of bacteria associated with gut of stingless bees. (PDF)

S3 Table The most abundant bacterial phyla in 6 stingless bee nests. (PDF)

S4 Table The most abundant bacterial genera in 6 stingless bee nests. *(Taxa in bracket were obtained from blast against NCBI database).
(PDF)

S1 File Accession numbers of nucleotide sequences in this study. (ZIP)

S2 File Measurements of the stingless bee specimens. (DOCX)

S1 Data Bacterial functional gene prediction by FAPROTAX. (XLSX)

S2 Data KEGG pathway entries with the top 50 ranking. (XLSX)

S3 Data Relative abundances of bacterial taxa in *T*. *pagdeni*. (XLSX)
