# Supplementary figures and images for: Microbial community profiling and culturing reveal functional groups of bacteria associated with Thai commercial stingless worker bees (Tetragonula pagdeni)
Source: PLoS One. 2023 Mar 1;18(3):e0280075. doi: 10.1371/journal.pone.0280075 (PMC9977063; doi:10.1371/journal.pone.0280075)

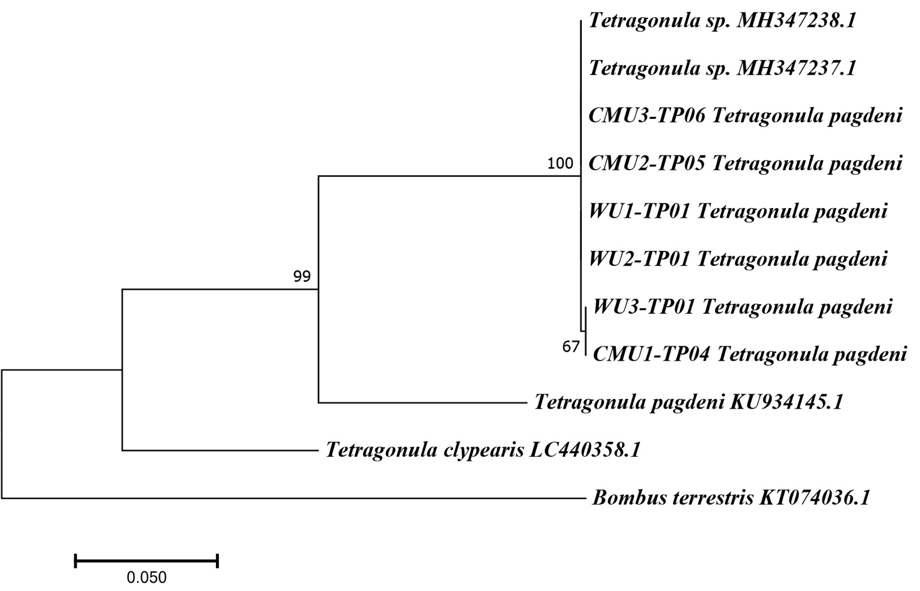

Supplement: S1 Fig — The tree was built using the Maximum Likelihood method. The sequences of B. terrestris–KT074036 was used as an outgroup to the tree. Numbers at each node represent bootstrap values as percentages of 1000 and only bootstrap greater than 70% are shown. (TIF) [file pone.0280075.s001.tif]

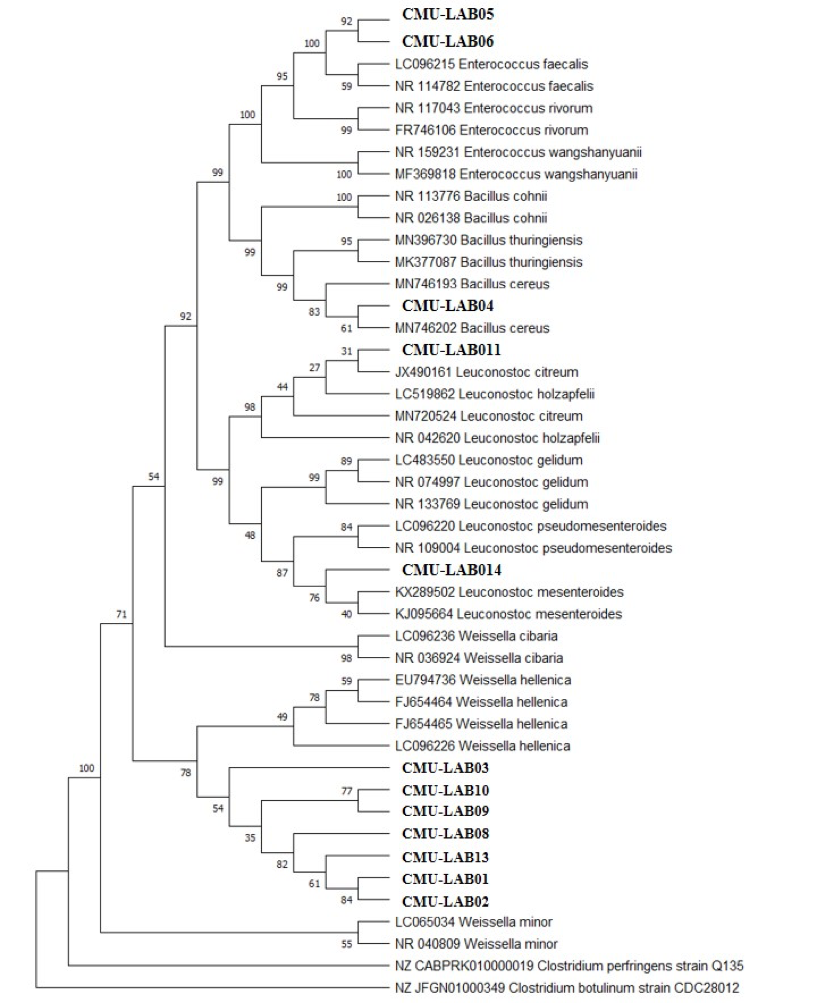

Supplement: S2 Fig — The sequences of Clostridium botulinum–JFGN01000349 was used as an outgroup. Numbers at each node represent bootstrap values as percentages and only bootstrap values greater than 70%. (TIF) [file pone.0280075.s002.tif]

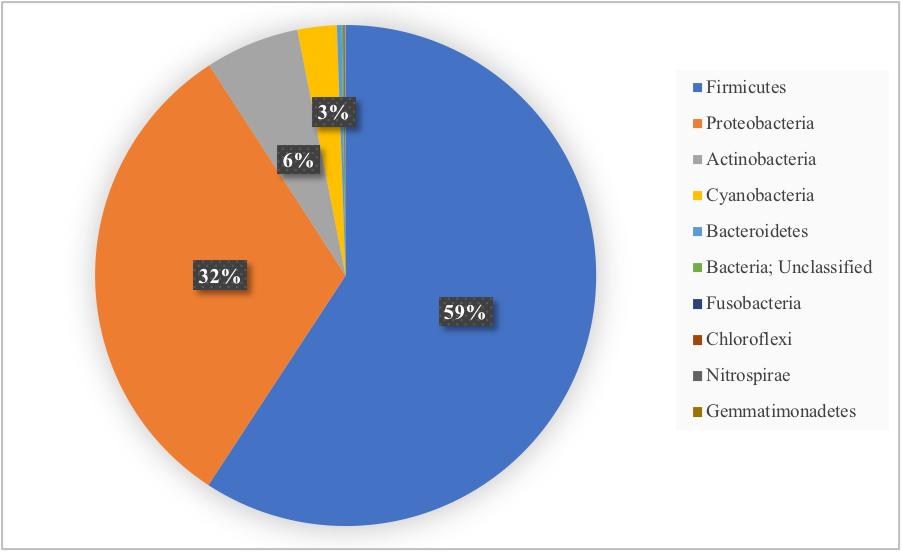

Supplement: S3 Fig — (TIF) [file pone.0280075.s003.tif]
