# Supplementary material for: Microbial community profiling and culturing reveal functional groups of bacteria associated with Thai commercial stingless worker bees (Tetragonula pagdeni)
Source: PLoS One. 2023 Mar 1;18(3):e0280075. doi: 10.1371/journal.pone.0280075 (PMC9977063; doi:10.1371/journal.pone.0280075)
Supplement: S1 Table — (PDF) [file pone.0280075.s004.pdf]

Supplementary Table S1 Material used in the phylogenetic analysis with the sample localities, and name of the collector

**Taxon**	**Sample name**	**Sample locality**	**Collector**	**260/280**	**Con(ng/ul)**	**Type**
Tetragonula pagdeni Smith	WU1-TP01	TH, Wat Umong CMP	C. Sinpoo	1.64	751.39	Dsdna
Tetragonula pagdeni Smith	WU2-TP02	TH, Wat Umong CMP	C. Sinpoo	1.79	825.89	Dsdna
Tetragonula pagdeni Smith	WU3-TP03	TH, Wat Umong CMP	C. Sinpoo	1.68	683.94	Dsdna
Tetragonula pagdeni Smith	CMU1-TP04	TH, Chaing Mai U CMP	C. Sinpoo	1.72	951.06	Dsdna
Tetragonula pagdeni Smith	CMU2-TP05	TH, Chaing Mai U CMP	C. Sinpoo	1.83	971.33	Dsdna
Tetragonula pagdeni Smith	CMU3-TP06	TH, Chaing Mai U CMP	C. Sinpoo	1.88	902.60	Dsdna
