# Supplementary material for: Microbial community profiling and culturing reveal functional groups of bacteria associated with Thai commercial stingless worker bees (Tetragonula pagdeni)
Source: PLoS One. 2023 Mar 1;18(3):e0280075. doi: 10.1371/journal.pone.0280075 (PMC9977063; doi:10.1371/journal.pone.0280075)
Supplement: S2 Table — (PDF) [file pone.0280075.s005.pdf]

Supplementary Table S2 Isolation and characterization of bacteria associated with stingless bees *T. pagdeni*

No.	Isolate	The most closely related with NCBI	Percent identity	Accession number of references
1	CMU-LAB01	*Weissella hellenica*	99.45	LC096226
2	CMU-LAB02	*Weissella hellenica*	99.45	LC096226
3	CMU-LAB03	*Weissella bombi*	98.39	NR136437
4	CMU-LAB04	*Bacillus cereus*	99.64	MN746202
5	CMU-LAB05	*Enterococcus faecalis*	98.66	LC096215
6	CMU-LAB06	*Enterococcus faecalis*	99.50	LC096215
7	CMU-LAB08	*Weissella hellenica*	99.17	LC096226
8	CMU-LAB09	*Weissella hellenica*	99.10	LC096226
9	CMU-LAB10	*Weissella hellenica*	98.54	FJ654464
10	CMU-LAB13	*Weissella hellenica*	98.95	FJ654465
11	CMU-LAB11	*Leuconostoc citreum*	99.72	JX490161
12	CMU-LAB14	*Leuconostoc mesenteroides*	99.97	KX289502
