# Supplementary material for: Microbial community profiling and culturing reveal functional groups of bacteria associated with Thai commercial stingless worker bees (Tetragonula pagdeni)
Source: PLoS One. 2023 Mar 1;18(3):e0280075. doi: 10.1371/journal.pone.0280075 (PMC9977063; doi:10.1371/journal.pone.0280075)
Supplement: S3 Table — (PDF) [file pone.0280075.s006.pdf]

Supplementary Table S3 The most abundant bacterial phyla in 6 stingless bee nests

Phylum	ST01	ST02	ST03	ST04	ST05	ST06	Sum	AV
Firmicutes	37985	17639	42477	32971	39200	39399	34945.17	59.229
Proteobacteria	18119	39199	12724	16740	13428	11706	18652.67	31.615
Actinobacteria	2245	1064	2110	5838	2964	7255	3579.33	6.067
Cyanobacteria	422	862	867	3264	3259	281	1492.50	2.530
Bacteroidetes	170	180	222	158	79	277	181.00	0.307
Bacteria; Unclassified	7	0	362	0	25	20	69.00	0.117
Fusobacteria	52	29	10	24	0	37	25.33	0.043
Chloroflexi	0	0	97	0	21	0	19.67	0.033
Nitrospirae	0	0	34	0	0	0	5.67	0.010
Gemmatimonadetes	0	0	12	0	19	0	5.17	0.009
TM7	0	27	4	0	0	0	5.17	0.009
Spirochaetes	0	0	12	0	0	18	5.00	0.008
Planctomycetes	0	0	20	0	5	0	4.17	0.007
Acidobacteria	0	0	16	0	0	7	3.83	0.006
[Thermi]	0	0	16	0	0	0	2.67	0.005
Chlamydiae	0	0	9	0	0	0	1.50	0.003
WPS-2	0	0	8	0	0	0	1.33	0.002
OD1	0	0	0	5	0	0	0.83	0.001
	59000	59000	59000	59000	59000	59000	59000.00	
