# Supplementary material for: Microbial community profiling and culturing reveal functional groups of bacteria associated with Thai commercial stingless worker bees (Tetragonula pagdeni)
Source: PLoS One. 2023 Mar 1;18(3):e0280075. doi: 10.1371/journal.pone.0280075 (PMC9977063; doi:10.1371/journal.pone.0280075)
Supplement: S4 Table — *(Taxa in bracket were obtained from blast against NCBI database). (PDF) [file pone.0280075.s007.pdf]

Supplementary Table S4 The most abundant bacterial genera in 6 stingless bee nests
*(Taxa in bracket were obtained from blast against NCBI database)

**Taxa**	**ST01**	**ST02**	**ST03**	**ST04**	**ST05**	**ST06**	**AV**	**sd**
*Lactobacillaceae**	27.34	16.68	19.93	43.11	41.00	35.50	30.59	11.02
Lactobacillus	23.07	8.46	46.89	2.93	17.05	26.48	20.81	15.52
*Halomonadaceae** *(Zymobacter, Carnimonas)*	20.42	49.81	1.76	21.94	10.76	15.87	20.09	16.29
Leuconostoc	12.67	4.43	3.21	6.38	6.12	1.08	5.65	3.96
Bombiscardovia	3.66	1.38	1.94	9.61	4.35	10.77	5.28	3.97
Saccharibacter	5.45	3.01	5.57	0.69	10.19	2.11	4.50	3.37
Pantoea	0.36	0.88	12.09	0.91	0.57	0.35	2.53	4.69
Streptophyta	0.72	1.46	1.44	5.52	5.22	0.46	2.47	2.28
*Xanthomonadaceae** *(Pseudomonas)*	0.02	11.01	0.00	0.00	0.01	0.01	1.84	4.49
Alkanindiges	0.57	0.11	0.09	3.54	0.39	0.00	0.78	1.37
Pediococcus	0.00	0.00	0.08	2.90	1.07	0.10	0.69	1.16
Lactobacillus	0.75	0.00	0.76	0.00	0.01	1.89	0.57	0.74
*Enterobacteriaceae** *(Klebsiella)*	3.07	0.02	0.00	0.04	0.13	0.09	0.56	1.23
other	1.91	2.76	6.25	2.44	3.13	5.30	3.63	1.74
