# Supplementary material for: Microbial community profiling and culturing reveal functional groups of bacteria associated with Thai commercial stingless worker bees (Tetragonula pagdeni)
Source: PLoS One. 2023 Mar 1;18(3):e0280075. doi: 10.1371/journal.pone.0280075 (PMC9977063; doi:10.1371/journal.pone.0280075)
Supplement: S2 File — (DOCX) [file pone.0280075.s009.docx]

|  | Structure | | |
| --- | --- | --- | --- |
| Number | Body length | head width | wing length |
| 1 | 4.51 | 1.75 | 1.08 |
| 2 | 4.33 | 1.75 | 1.06 |
| 3 | 4.56 | 1.75 | 1.08 |
| 4 | 4.85 | 1.75 | 1.14 |
| 5 | 4.58 | 1.72 | 1.12 |
| 6 | 4.45 | 1.74 | 1.13 |
| 7 | 4.37 | 1.72 | 1.08 |
| 8 | 4.59 | 1.73 | 1.04 |
| 9 | 4.54 | 1.74 | 1.07 |
| 10 | 4.69 | 1.72 | 1.13 |
| 11 | 4.79 | 1.75 | 1.13 |
| 12 | 4.65 | 1.72 | 1.08 |
|  |  |  |  |
| Average±SD | 4.576 | 1.737 | 1.095 |
|  | 0.155 | 0.014 | 0.033 |

Measurements of stingless bee specimens
